# Predictive value of MRI-detected tumor deposits in locally advanced rectal cancer

**DOI:** 10.3389/fonc.2023.1153566

**Published:** 2023-08-21

**Authors:** Baohua Lv, Xiaojuan Cheng, Yanling Cheng, Xue Kong, Erhu Jin

**Affiliations:** ^1^ Department of Radiology, Taian City Central Hospital, Qingdao University, Tai’an, China; ^2^ Department of Radiology, Beijing Friendship Hospital, Capital Medical University, Beijing, China; ^3^ Clinical Skills Center, Taian Central Hospital, Tai’an, China; ^4^ Respiratory Department, Shandong Second Rehabilitation Hospital, Tai’an, China

**Keywords:** rectal cancer, tumor deposits, lymph node metastasis, extramural vascular invasion, distant metastasis

## Abstract

**Background:**

Although tumor deposits (TDs) are not the same as lymph nodes, the prognosis of patients with TDs is similar or worse than that of patients with metastatic lymph nodes. TDs are mostly assessed by the histology of samples after surgery, thus, not helpful for preoperative treatment strategies. The primary objective of this study was to detect TDs by MRI and evaluate its predictive value.

**Materials and methods:**

A total of 114 patients with rectal cancer were retrospectively analyzed. Clinicopathological and MRI data mainly including MRI- detected TDs (mTDs), tumor border configuration (TBC) on MRI, MRI-detected extramural vascular invasion (mEMVI), MRI-detected lymph node metastasis (mLN), MRI T stage, MRI N stage, the range of rectal wall involved by the tumor, peritoneal reflection invasion, tumor length, tumor location, cord sign at the tumor edge, nodular protrusion at the tumor edge, maximal extramural depth and pathology-proven lymph node involvement (pLN) were evaluated. The correlation of MRI factors with postoperative distant metastasis (PDM) and pLN were analyzed by univariate analysis and multivariate logistic regression analysis, and nomograms were established based on the latter. The diagnostic efficiency was evaluated by the receiver operating characteristic curve (ROC) and area under the curve (AUC).

**Results:**

A total of 38 cases of pLN, 13 of PDM and 17 of pathology-proven TDs (pTDs) were found. Ten cases of PDM and 22 cases of pLN in 30 mTDs cases were also found. Chi-square test showed that mTDs, mLN, TBC, mEMVI, MRI T stage, nodular protrusion, cord sign, maximal extramural depth and peritoneal reflection invasion were correlated with PDM and pLN (*P*<0.05). mTDs and peritoneal reflection invasion were independent risk factors for PDM (odds ratio: 10.15 and 8.77, *P*<0.05), mTDs and mLN were independent risk factors for pLN (odds ratio: 5.50 and 5.91, *P*<0.05), and Hosmer-Lemeshow test showed that the results of two models were not statistically significant, suggesting that the fit was good. On this basis, two nomograms for predicting PDM and pLN were confirmed by Bootstrap self-sampling, and the C-indices of the two nomograms were 0.837 and 0.817, respectively. The calibration curves and ROC curves of the two nomograms showed that the correlation between the predicted and the actual incidence of PDM and pLN was good. The DeLong test showed that the predictive efficiency of the nomogram in predicting pLN was better than that of mLN (*P=*0.0129).

**Conclusion:**

mTDs are a risk factor for PDM and lymph node metastasis. The two nomograms based on mTDs showed a good performance in predicting PDM and lymph node metastasis, possessing a certain clinical value.

## 1 Introduction

Colorectal cancer is one of the four deadliest cancers worldwide, with an incidence ranking third and mortality ranking second. More than 1.9 million patients were diagnosed with colorectal cancer worldwide in 2020, 940,000 people died of rectal cancer (1), and more than 50% of the new cases and deaths were caused by rectal cancer. Colorectal cancer incidence is increasing in developing countries, and the number of new cases is expected to increase to 2.5 million globally by 2035 (2, 3). Postoperative distant metastasis (PDM), recurrence, lymph node metastasis, venous infiltration and perineural infiltration are important factors affecting the prognosis of rectal cancer patients (4–6). At present, tumor deposits (TDs) have been incorporated into TNM, but numerous studies showed that TDs are not the same as lymph nodes and the prognosis of patients with TDs is similar or worse than that of patients with metastatic lymph nodes (7–10). TDs are mostly assessed by the histology of samples after surgery, thus, not helpful for preoperative treatment strategies. At present, few image reports are available on TDs. MRI, especially high-resolution MRI (HRMRI), can clearly show some special nodules around the rectum thanks to its soft tissue resolution, with significantly different morphology and signal from common lymph nodes. In addition, MRI allows a comprehensive multiplanar assessment of the relationship between these particular nodes and the surrounding structures such as blood vessels in oblique axial, oblique coronal and sagittal planes, assessment that is more difficult to perform by pathological histology. Therefore, the primary objective of this study was to detect TDs by MRI and evaluate its predictive value.

## 2 Materials and methods

### 2.1 Patients

**Figure 1:**
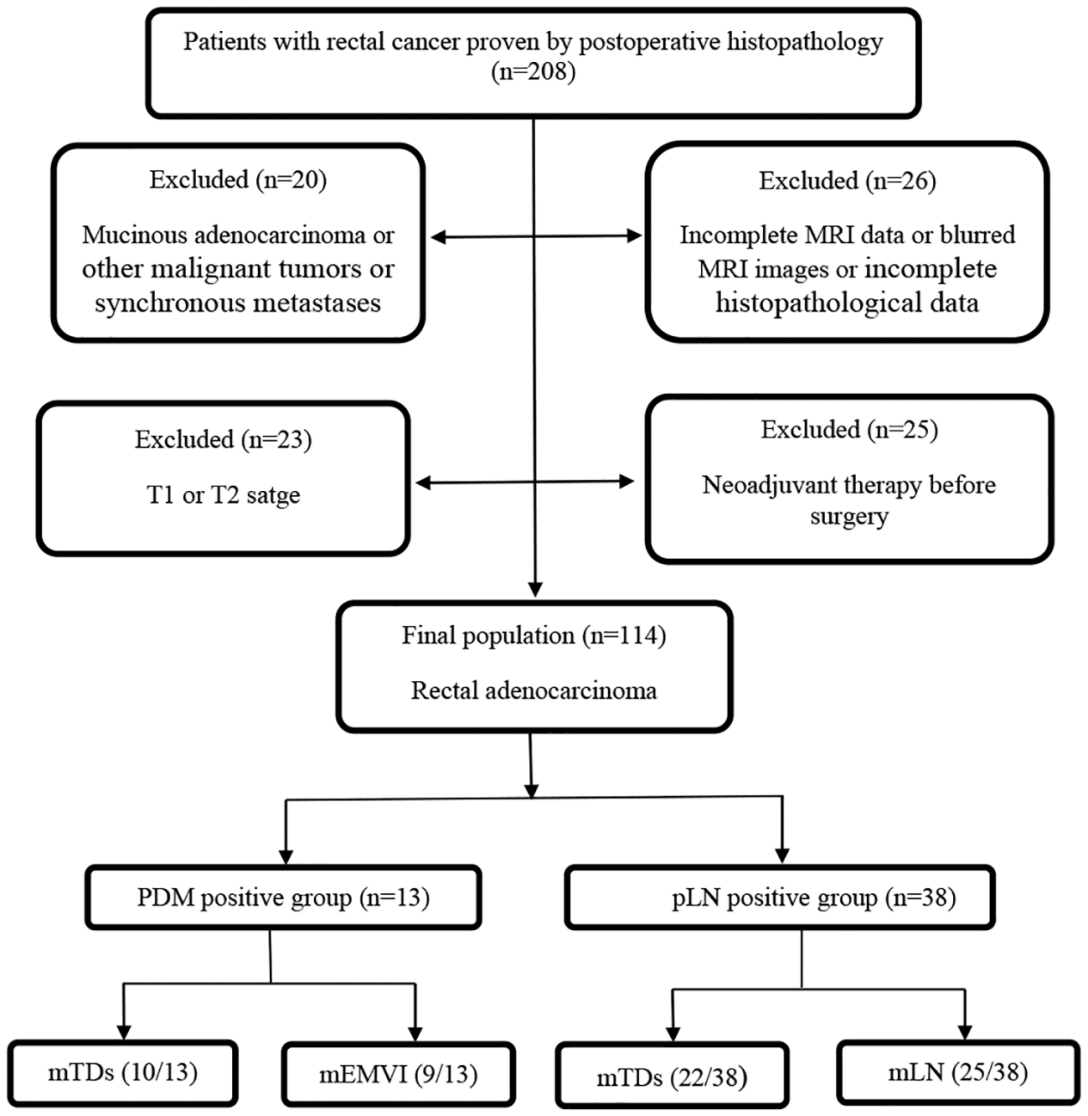
Study flow diagram. PDM, postoperative distant metastases; pLN, pathology-proven lymph node involvement; mTDs, MRI-detected tumor deposits; mEMVI, MRI-detected extramural vascular invasion; mLN, MRI-detected lymph node involvement.

This study was approved by the Ethics Committee of Beijing Friendship Hospital, and individual consent was not required for this retrospective analysis. The medical records and MRI data of 208 patients who were subjected to a total mesorectal excision in the above hospital from 2014 to 2019 were retrospectively analyzed. The mass removed from all patients was confirmed as rectal cancer by postoperative histopathology. All MRI scans of rectal cancer patients were performed before surgery. The exclusion criteria were the following: (i) patients with blurred preoperative MRI images or incomplete MRI sequences (11 cases); (ii) patients receiving neoadjuvant therapy before surgery (25 cases); (iii) patients with other malignant tumors or synchronous metastases (18 cases); (iv) patients with incomplete histopathological data (15 cases); (v) patients with rectal cancer in T1 and T2 stages (23 cases); (vi) patients with mucinous adenocarcinoma (2 cases) because of their specific biological behavior. Finally, 114 cases were enrolled (**Figure 1**). with 69.3% (n=79) of patients undergoing the Dixon procedure, 19.3% (n=22) undergoing the Miles procedure, 7.0% (n=8) undergoing Hartmann’s procedure, and 4.4% (n=5) undergoing transanal total mesorectal excision (TaTME).

### 2.2 MRI technique

MRI was performed using a 3.0T system (Signa Excite HD 3.0T, GE Healthcare, Milwaukee, WI, USA) equipped with 8-channel body surface coil. Patients were advised to eat light food 24 hours before the scan according to the daily volume and defecation in time. Before the MR scan, enema was cleaned, and antispasmodic drugs were not injected. Pulse sequences were observed by fast spin-echo sagittal high-resolution T2WI (HRT2WI) with a thickness of 3 mm, an intersection gap of 0.5 mm, and a TR/TE of 4,000 ms/102 ms, in the absence of fat saturation. The matrix size was 384×360. The ETL and NEX were 16 and 4, respectively. The oblique axial HRT2WI was perpendicular to the rectal wall and covered the entire tumor in the absence of fat saturation with a thickness of 3 mm, an intersection gap of 0.5 mm, a TR/TE of 4,900 ms/96 ms, matrix size of 320X256 and FOV of 20X22 cm and resolution ratio 0.87 mm X 0.78 mm. When the scan at the oblique coronal HRT2WI plane was performed, the scanning plane was parallel to the intestinal wall of the lesion area and covered the whole tumor.

A LAVA/LAVA-XV sequence was performed for dynamic contrast-enhanced MRI (DCE-MRI) in the presence of fat saturation, a thickness of 3 mm, TR 3.6 ms, TE 1.38 ms, FOV of 36×36 cm, matrix size of 256×192, flip angle of 15°, and 9 consecutive phases. Gd-DTPA (0.1 mmol/kg, Magnevist, Bayer Schering, Germany) was intravenously injected at a rate of 2 ml/s, followed by a saline flush before enhanced sequencing.

### 2.3 Imaging interpretation

The MRI images of all patients were retrospectively analyzed on oblique axial, sagittal, oblique coronal HRT2WI images and gadolinium enhanced T1WI by two radiologists with 8 and 16 years of experience in abdominal MRI and familiar with the diagnostic criteria. In case of disagreement, a third experienced gastrointestinal radiologist was involved and consensus was reached by discussion. All radiologists were blind to patients’ PDM as well as to their pathological results of lymph node.

#### 2.3.1 Evaluation of multiple MRI signs

**Figure 2:**
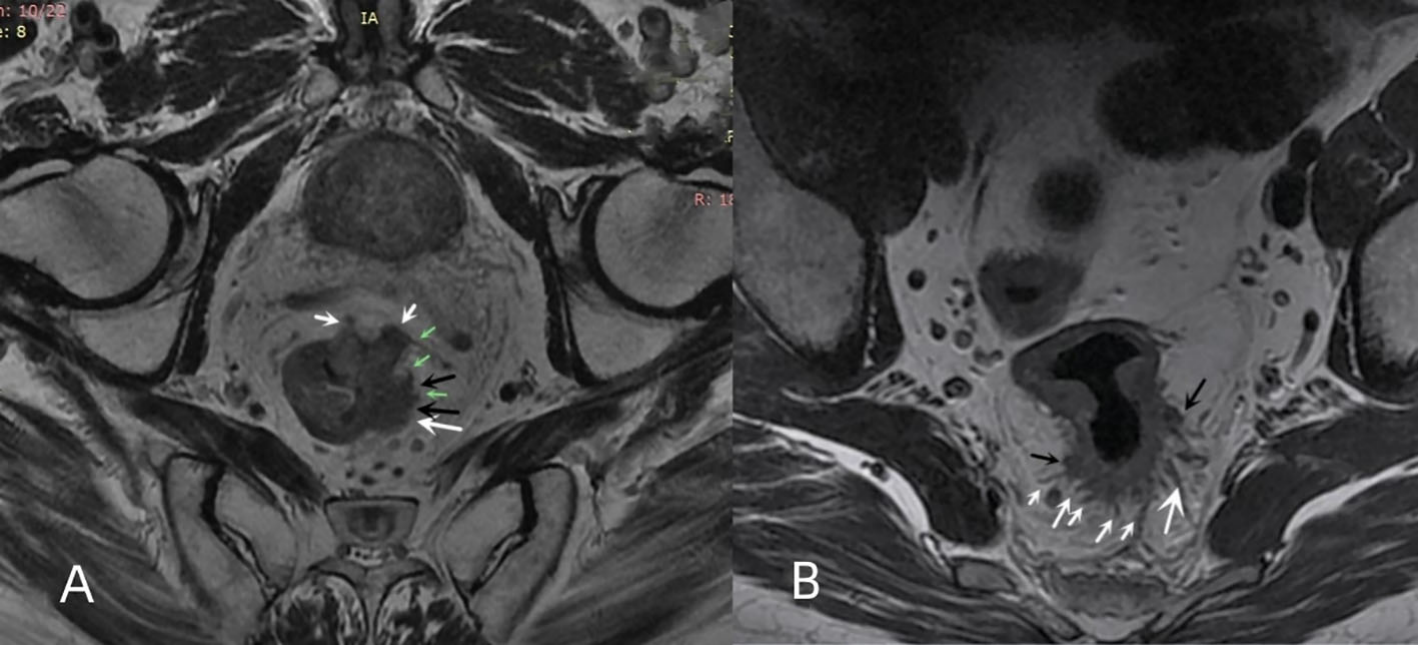
Multiple nodular protrusions and cords of iTBC. **(A)** oblique axial HRT2WI imaging of a 67-year-old male patient with rectal cancer showing multiple irregular nodular protrusions (white arrows) appearing at the margin of the primary rectal tumor. Some nodules had lobulated edges (black arrows), and some nodules had irregular cords (green arrows). **(B)** oblique axial HRT2WI imaging of a 43-year-old male patient with rectal cancer showing multiple cords (white arrows) of uneven thickness and two irregularly shaped nodular protrusions (black arrows) appearing at the posterior margin of the primary tumor.

The MRI tumor border morphology was divided into infiltrating tumor border configuration (iTBC) and pushing tumor border configuration (pTBC) according to the histopathological TBC (11, 12). iTBC was mainly characterized by the following aspects: (i) presence of one or more irregularly shaped nodular projections with hairy or lobulated margins in the margin of the primary tumor (**Figure 2A**); (ii) The presence of multiple rough cords with uneven thickness on the edge of the primary tumorr (**Figure 2B**). pTBC was mainly characterized by the following aspects: the margins of the primary tumor or the single nodular projection at the tumor margin were clear and smooth, or the cords at the tumor margin were uniformly thick and well-defined.

**Figure 3:**
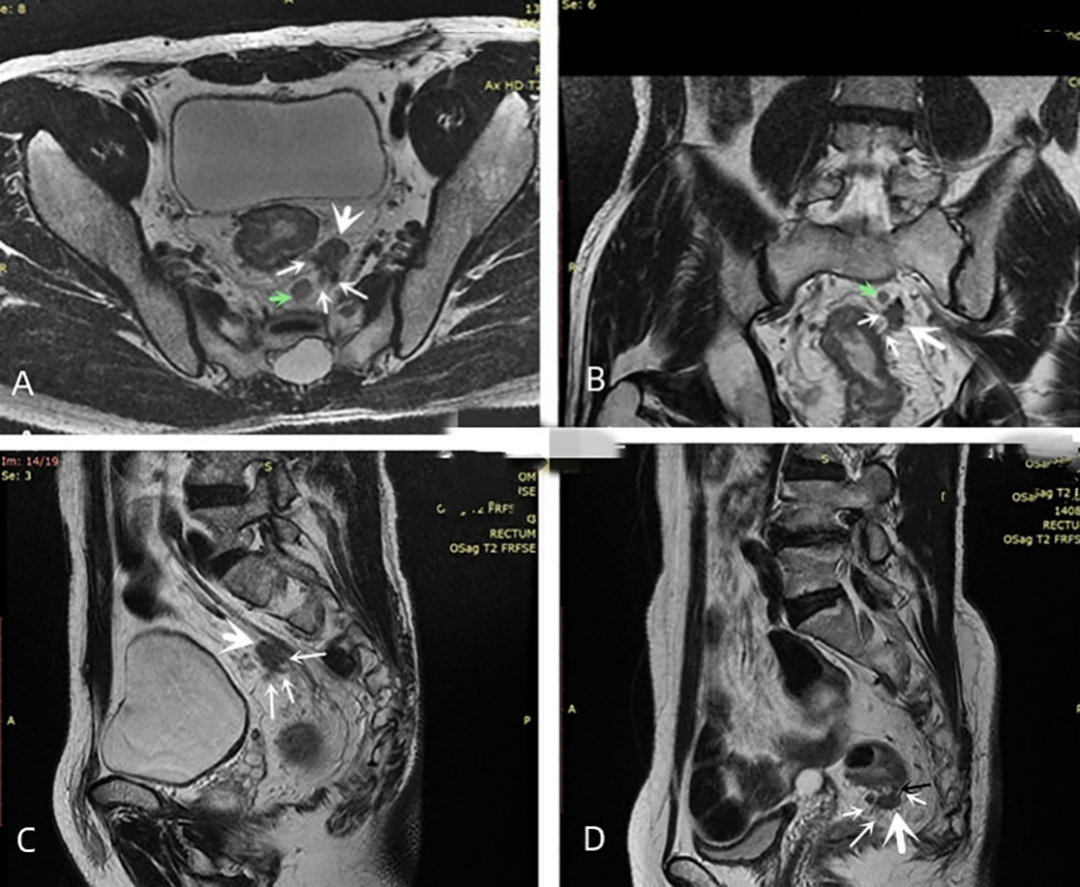
Diagram of mTDs. **(A–C)** oblique axial, oblique coronal and sagittal images showing the same irregularly shaped nodule (large white arrow) with multiple irregularly dilated veins (multiple thin white arrows) at the edge of this nodule entering the nodule. The signal intensity on T2-weighted images of both the nodule and the surrounding invaded vessels was slightly lower than that of the adjacent lymph node (green arrow). **(D)** the narrow base between the nodule (black arrow) was an invaded vessel, and multiple vessels surrounding the nodule were present and entered inside it.

mEMVI was evaluated according to Smith’s five level scoring system (13). (i) Score 0: the tumor did not penetrate the muscularis propria of the rectal wall. (ii) Score 1: the tumor invaded the muscularis propria of the rectal wall, but no blood vessel formation was found around the tumor. (iii) Score 2: the tumor invaded the muscularis propria of the rectal wall, but the diameter and signal of the peritumoral blood vessels were normal, and no abnormal blood vessels were observed by gadolinium-enhanced T1WI. (iv) Score 3: the tumor invaded the muscularis propria of the rectal wall and intestinal wall, and the peritumoral vascular diameter was slightly irregularly dilated, or a tumor with moderate intensity signal was observed in the lumen. The lumen of the diseased vessel was slightly dilated, as observed by gadolinium enhanced T1WI, and abnormal signals similar to tumor enhancement appeared in the lumen. (v) Score 4: the tumor penetrated the muscularis propria of the rectum wall, and the lumen of one or more blood vessels around the tumor were irregularly expanded. Tumor tissue was present in the lumen, and the signal intensity was moderate. A significant irregular dilation of the lumen of one or more vessels in the tumor was observed, with tumor-like enhancement of the abnormal signals in their lumens was found by gadolinium-enhanced T1WI. (vi) 3 or 4 score: the irregular nodules in the mesenteric fascia involved the adjacent small vessels, and the lumen was slightly dilated (**Figure 3**). The mEMVI negative had a score of 0 to 2, while the mEMVI positive had a score of 3 to 4.

**Figure 4:**
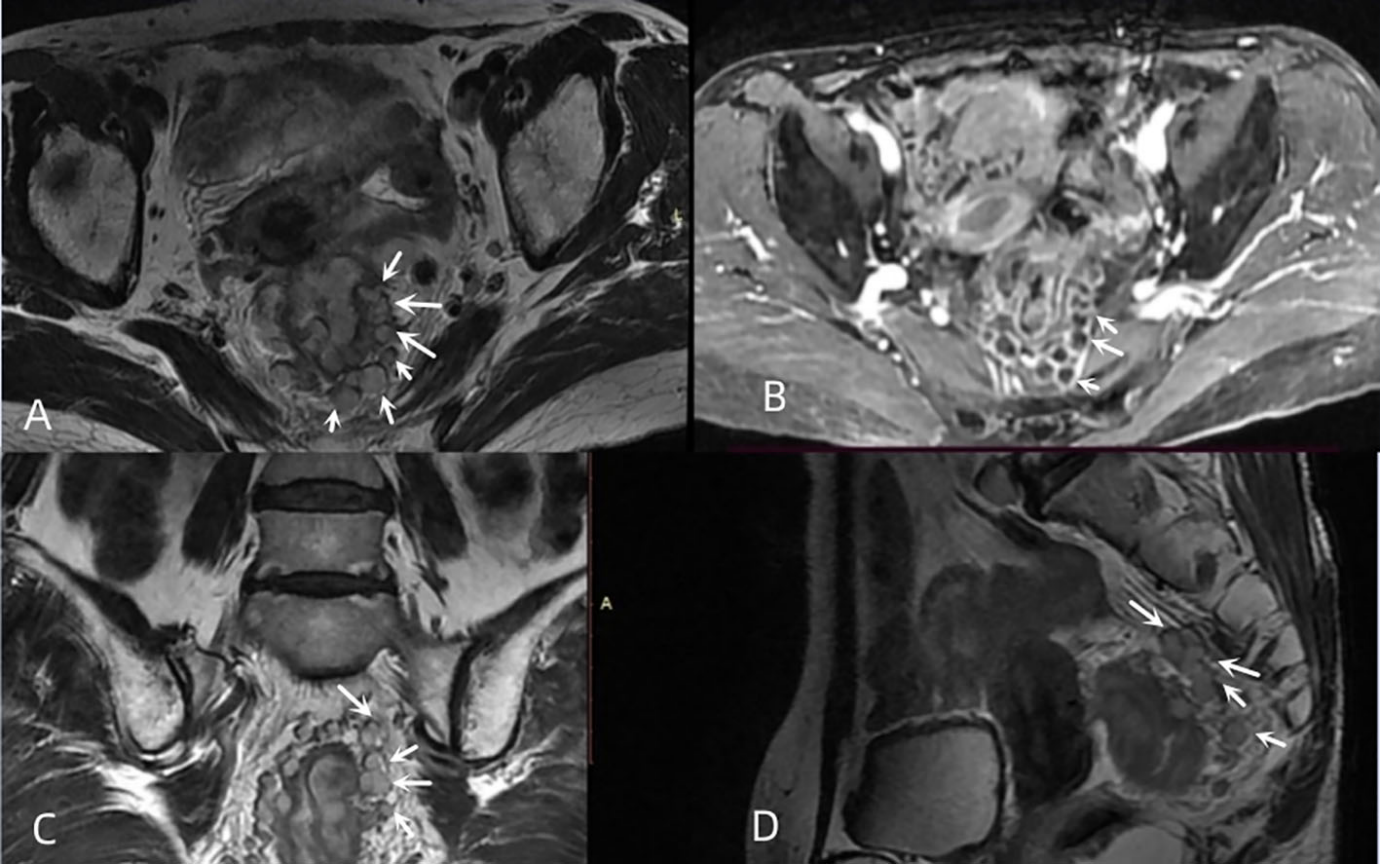
Multiple nodal fusion of mTDs. **(A, C, D)** oblique axial, oblique coronal, and sagittal images of a 61-year-old female with rectal cancer showing multiple nodules (white arrows) arranged in clusters around the rectal tumor, partially fused into a mass. **(B)** contrast-enhanced T1WI showing multiple fused nodules in a circular pattern with a significant enhancement (white arrows).

mTDs were observed by oblique axial, oblique coronal and sagittal HRT2WI and mainly included the following conditions: (i) the morphology of a nodule in the pelvis, which was discontinuously or narrowly basally connected to the primary tumor of the rectal cancer, was irregular or round or elliptical, and the nodule had a slightly lower signal intensity on T2-weighted images. The high spatial resolution of the T2-weighted imaging performed in three orthogonal planes confirmed that the neighboring veins invaded the nodule, with an abnormal morphology or signal changes of the vessels (**Figure 3**); (ii) when no clear vessels invading the nodule were present, the multiple irregular nodules were clustered and some nodules were fused (**Figure 4**).

Intrapelvic mLN was mainly evaluated by oblique axial, oblique coronal and sagittal HRT2WI combined with DWI. The nodules in the pelvis consistent with mTDs were initially excluded, and mLN was diagnosed as one of the following three conditions (14): (i) nodules with a short-axis diameter greater than 10 mm. (ii) nodules with a short-axis diameter of 5 to 10 mm with irregular margins (burr or lobulated or fuzzy) and internal signal inhomogeneity. (iii) nodules with short-axis diameter less than 5 mm that had irregular margins (burr or lobulation or gross blurring), accompanied by internal signal inhomogeneity and high signal on high b-value DWI.

#### 2.3.2 Evaluation of other MRI parameters

The length from the most distant point of the tumor to the rectal lamina propria in the area where the tumor was located on the oblique axial HRT2WI was defined as maximal extramural depth and was divided into two groups: < 5 mm and ≥ 5mm. Tumor length was the length measured at multiple points between the upper and lower borders of the tumor along the sagittal axis of the diseased bowel and was divided into two groups: < 5 cm and ≥ 5 cm. The tumor was classified as middle and upper rectal cancer when the distance between the lower border of the rectal tumor and the anal verge was more than 5 cm, while it was classified as lower rectal cancer when the distance was less than 5 cm. The range of the rectal wall involved in the tumor was estimated on oblique axial HRT2WI and was divided into three groups: ≤ 1/3, 1/3-2/3 and ≥ 2/3. MRI T and N stage of rectal cancer was done using the American Joint Committee on Cancer (AJCC, eighth edition).

### 2.4 Clinicopathological assessment and management protocol for rectal cancer

The samples collected during surgery were stored in 10% formalin for more than 48 hours and cut laterally and perpendicular to the long axis of the rectum with a thickness of 3 µm. TDs and local lymph node (when involved) were collected and analyzed by histopathological examination. Histopathology was performed by a pathologist with extensive experience in colorectal pathology.

Initial evaluation of suspected rectal cancer patients involves physical examinations, such as digital rectal examination, laboratory tests including CEA measurement, imaging evaluations such as CT and MRI scans, or endoscopy to confirm the diagnosis and perform preoperative assessment. Treatment plans are formulated through a multidisciplinary approach known as multi-disciplinary treatment (MDT). For rectal cancer cases with a distance from the anal verge of <12cm on MRI evaluation, preoperative neoadjuvant therapy is recommended. Patients with T1-2N0M0 stage or contraindications for chemotherapy and radiation therapy are generally advised to undergo direct surgery. For potentially resectable rectal cancer cases with T3 and/or N+ involvement, preoperative neoadjuvant therapy is typically recommended. Patients with T4 or locally advanced unresectable disease should undergo preoperative chemoradiotherapy. Early-stage rectal cancer (cT1N0M0) may be eligible for local excision procedures such as transanal endoscopic microsurgery (TEM). Radical surgery is recommended for cT2-4N0-2M0 rectal cancer cases. For patients with stage II with high-risk features and stage III rectal cancer, adjuvant chemoradiotherapy and other comprehensive treatments are recommended postoperatively. Regular follow-up is recommended for postoperative rectal cancer patients. Due to constraints imposed by local economic conditions and socio-economic factors, a minority of patients were able to undergo preoperative neoadjuvant chemoradiotherapy.

### 2.5 Follow up

A two-year follow up after surgery was performed on all patients. Outpatient follow-up was every 3 months; pelvic MRI, liver ultrasound and serum tumor markers were monitored every 6 months; postoperative colonoscopy was performed once a year; high-risk patients (those with positive extramural vascular invasion, positive circumferential margins, regional lymph node metastasis, and low-grade rectal cancer) were annually monitored by performing chest and whole abdomen computed tomography (CT) enhancement scans and MRI scan of the pelvis. The inclusion of postoperative distant metastases should meet the following criteria: (i) histological examination confirming the pathological diagnosis. (ii) Positive PET-CT findings. (iii) CT, MRI, or endoscopic ultrasonography revealing the typical manifestation of metastases.

### 2.6 Statistical analysis

Statistical analysis was performed using IBM SPSS 26.0, MedCalc and R (R 4.2.1) software. The correlation between clinicopathological factors and MRI signs with PDM and pLN was analyzed by Chi-square test and ANOVA. The variables with p<0.05 in the univariate analysis were included in a binary logistic regression model. In this model, PDM and pLN were used as dependent variables, while mTDs, mLN, and other variables were used as independent variables. The binary logistic stepwise regression (Forward: LR) method was utilized to analyze the impact of mTDs on PDM and pLN, after adjusting for confounding factors. The nomograms for predicting PDM and pLN were constructed on the basis of multivariate logistic regression analysis, and the C-indices of the nomograms were calculated to determine the differentiation of the models. The predictive performance of MRI signs for PDM and pLN was analyzed by plotting the receiver operating characteristic (ROC) curves. The comparisons among the ROC curves of the indicators were performed by the DeLong test. Interobserver agreement and agreement between pathological findings and imaging assessment results were performed using the Kappa test (Kappa > 0.75 indicated good consistency, 0.40 > Kappa ≤ 0.75 indicated moderate consistency, and Kappa ≤ 0.40 indicated poor consistency). A value of *P* < 0.05 was considered statistically significant.

## 3 Results

### 3.1 Patient characteristics

A total of 114 patients (76 males and 38 females) with rectal cancer were enrolled in this study. The mean age of these patients was 64.18 ± 10.06 yrs, while that for the male and female was 65.26 ± 10.78 yrs and 62.03 ± 11.45 yrs, respectively. Demographic and MRI data are listed in **Tables 1** and **2**.

**Table 1:** Characteristics of MRI and clinicopathological data.

Variable	n=114	PDM		pLN		Local recurrence	
		Negative (n=101) n(%)	Positive (n=13) n(%)	Negative (n=76) n(%)	Positive (n=38) n(%)	Negative (n=102) n(%)	Positive (n=12) n(%)
Age (year)	64.18 ± 11.06	64.24 ± 10.94	63.77 ± 12.41	64.75 ± 11.20	63.05 ± 10.84	64.02 ± 10.92	65.58 ± 12.67
Gender							
Male	76(66.7)	68(67.3)	8(61.5)	52(68.4)	24(63.2)	66(64.7)	10(83.3)
Female	38(33.3)	33(32.7)	5(38.5)	24(31.6)	14(36.8)	36(35.3)	2(16.7)
Range of rectal wall involved							
≤1/3	3(2.6)	3(3.0)	0(0)	3(3.9)	0(0)	3(2.9)	0(0)
1/3–2/3	39(34.2)	38(37.6)	1(7.7)	27(35.5)	12(31.6)	37(36.3)	2(16.7)
≥2/3	72(63.2)	60(59.4)	12(92.3)	46(60.5)	26(68.4)	62(60.8)	10(83.3)
TBC							
iTBC	47(41.2)	37(36.6)	10(76.9)	22(28.9)	25(65.8)	39(38.2)	8(66.7)
pTBC	67(58.8)	64(63.4)	3(23.1)	54(71.1)	13(34.2)	63(61.8)	4(33.3)
mEMVI	35 (30.7)	26(25.7)	9(69.2)	14(18.4)	21(55.3)	27(26.5)	8(66.7)
Pathological EMVI	36(31.6)	30(29.7)	6(46.2)	14(18.4)	22(57.9)	31(30.4)	5(41.7)
Tumor location							
Upper-middle	82 (71.9)	73(72.3)	9 (69.2)	52(68.4)	30(78.9)	73(71.6)	9(75.0)
Lower	32 (28.1)	28(27.7)	4 (30.8)	24(31.6)	8(21.1)	29(28.4)	3(25.0)
Peritoneal reflection invasion	9(7.9)	4(4.0)	5(38.5)	3(3.9)	6(15.8)	5(4.9)	4(33.3)
Tumor length (cm)							
≤5	62(54.4)	57(56.4)	5(38.5)	40(52.6)	22(57.9)	57(55.9)	5(41.7)
>5	52(45.6)	44(43.6)	8(61.5)	36(47.4)	16(42.1)	45(44.1)	7(58.3)
mTDs	30(26.3)	20(19.8)	10(76.9)	8(10.5)	22(57.9)	27(26.5)	3(25.0)
mLN	36(31.6)	26(25.7)	10(76.9)	11(14.5)	25(65.8)	31(30.4)	5(41.7)
Maximal extramural depth (mm)							
<5	73(64.0)	71(70.3)	2(15.4)	58(76.3)	15(39.5)	70(68.6)	3(25.0)
≥5	41(36.0)	30(29.7)	11(84.6)	18(23.7)	23(60.5)	32(31.4)	9(75.0)
Cord sign	42(36.8)	32(31.7)	10(76.9)	20(26.3)	22(57.9)	34(33.3)	8(66.7)
Nodular protrusion	38(33.3)	30(29.7)	8(61.5)	16(21.1)	22(57.9)	31(30.4)	7(58.3)
Pelvic side wall nodes on MRI	12(10.5)	9(8.9)	3(23.1)	4(5.3)	8(21.1)	10(9.8)	2(16.7)
pTDs	17(14.9)	13(12.9)	4(30.8)	5(6.6)	12(31.6)	13(12.7)	4(33.3)
MRI-detected CRM	16(14.0)	11(10.9)	5(38.5)	6(7.9)	10(26.3)	12(11.8)	4(33.3)
Pathological CRM	10(8.8)	5(5.0)	5(38.5)	4(5.3)	6(15.8)	6(5.9)	4(33.3)
MRI T stage							
T3	98(86.0)	91(90.1)	7(53.8)	69(90.8)	29(76.3)	92(90.2)	6(50.0)
T4	16(14.0)	10(9.9)	6(46.2)	7(9.2)	9(23.7)	10(9.8)	6(50.0)
Pathological T stage							
T3	106(93.0)	96(95.0)	10(76.9)	74(97.4)	32(84.2)	96(94.1)	10(83.3)
T4	8(7.0)	5(5.0)	3(23.1)	2(2.6)	6(15.8)	6(5.9)	2(16.7)
MRI N stage							
N0	78(68.4)	75(74.3)	3(23.1)	65(85.5)	13(34.2)	71(69.6)	7(58.3)
N1	21(18.4)	16(15.8)	5(38.5)	7(9.2)	14(36.8)	18(17.6)	3(25.0)
N2	15(13.2)	10(9.9)	5(38.5)	4(5.3)	11(28.9)	13(12.7)	2(16.7)
Pathological N stage							
N0	76(66.7)	70(69.3)	6(46.2)	76(100)	0(0)	69(67.6)	7(58.3)
N1	28(24.6)	24(23.8)	4(30.8)	0(0)	28(73.7)	25(24.5)	3(25.0)
N2	10(8.8)	7(6.9)	3(23.1)	0(0)	10(26.3)	8(7.8)	2(16.7)
Pathological type							
Ulcerative adenocarcinoma	95 (83.3)	82(81.2)	13(100)	62(81.6)	33(86.8)	84(82.4)	11(91.7)
Elevated adenocarcinoma	19 (16.7)	19 (18.8)	0 (0)	14(18.4)	5(13.2)	18(16.6)	1(8.3)

**Table 2:** Results of univariate analysis.

Variable	PDM				pLN		
	n=114	Negative (n=101) n (%)	Positive (n=13) n (%)	P value	Negative (n=76) n (%)	Positive (n=38) n (%)	P value
Age (year)				0.947			0.669
<60	36(31.6)	32(31.7)	4(30.8)		23(30.3)	13(34.2)	
≥60	78(68.4)	69(68.3)	9(69.2)		53(69.7)	25(65.8)	
Gender				0.677			0.574
Male	76(66.7)	68(67.3)	8(61.5)		52(68.4)	24(63.2)	
Female	38(33.3)	33(32.7)	5(38.5)		24(31.6)	14(36.8)	
Range of rectal wall involved				0.70*			0.40*
≤1/3	3(2.6)	3(3.0)	0(0)		3(3.9)	0(0)	
1/3–2/3	39(34.2)	38(37.6)	1(7.7)		27(35.5)	12(31.6)	
≥2/3	72(63.2)	60(59.4)	12(92.3)		46(60.5)	26(68.4)	
TBC				0.005			<0.001
iTBC	47(41.2)	37(36.6)	10(76.9)		22(28.9)	25(65.8)	
pTBC	67(58.8)	64(63.4)	3(23.1)		54(71.1)	13(34.2)	
mEMVI				<0.001			<0.001
Positive	35 (30.7)	26(25.7)	9(69.2)		14(18.4)	21(55.3)	
Negative	79(69.3)	75(74.3)	4(30.8)		62(81.6)	17(44.7)	
Tumor location				1.00			0.238
Upper-middle	82(71.9)	73(72.3)	9 (69.2)		52(68.4)	30(78.9)	
Lower	32(28.1)	28(27.7)	4 (30.8)		24(31.6)	8(21.1)	
Peritoneal reflection invasion				<0.001			0.027
Positive	9(7.9)	4(4.0)	5(38.5)		3(3.9)	6(15.8)	
Negative	105(92.1)	97(96.0)	8(61.5)		73(96.1)	32(84.2)	
Tumor length (cm)				0.221			0.595
≤5	62(54.4)	57(56.4)	5(38.5)		40(52.6)	22(57.9)	
>5	52(45.6)	44(43.6)	8(61.5)		36(47.4)	16(42.1)	
mTDs				<0.001			<0.001
Positive	30(26.3)	20(19.8)	10(76.9)		8(10.5)	22(57.9)	
Negative	84(73.7)	81(80.2)	3(23.1)		68(89.5)	16(42.1)	
mLN				<0.001			<0.001
Positive	36(31.6)	26(25.7)	10(76.9)		11(14.5)	25(65.8)	
Negative	78(68.4)	75(74.3)	3(23.1)		65(85.5)	13(34.2)	
Maximal extramural depth				<0.001			<0.001
<5mm	73(64.0)	71(70.3)	2(15.4)		58(76.3)	15(39.5)	
≥5mm	41(36.0)	30(29.7)	11(84.6)		18(23.7)	23(60.5)	
Cord sign				<0.001			<0.001
Positive	42(36.8)	32(31.7)	10(76.9)		20(26.3)	22(57.9)	
Negative	72(63.2)	69(68.3)	3(23.1)		56(73.7)	16(42.1)	
Nodular protrusion				0.022			<0.001
Positive	38(33.3)	30(29.7)	8(61.5)		16(21.1)	22(57.9)	
Negative	76(66.7)	71(70.3)	5(38.5)		60(79.9)	16(42.1)	
pTDs				0.09			<0.001
Positive	17(14.9)	13(12.9)	4(30.8)		5(6.6)	12(31.6)	
Negative	97(85.1)	88(87.1)	9(69.2)		71(93.4)	26(68.4)	
MRI T stage				<0.001			0.036
T3	98(86.0)	91(90.1)	7(53.8)		69(90.8)	29(76.3)	
T4	16(14.0)	10(9.9)	6(46.2)		7(9.2)	9(23.7)	
MRI N stage				<0.001			<0.001
N0	78(68.4)	75(74.3)	3(23.1)		65(85.5)	13(34.2)	
N1	21(18.4)	16(15.8)	5(38.5)		7(9.2)	14(36.8)	
N2	15(13.2)	10(9.9)	5(38.5)		4(5.3)	11(28.9)	

PDM, postoperative distant metastases; pLNm, pathology-proven lymph node involvement; mEMVI, MRI-detected extramural vascular invasion; TBC: tumor border configuration; iTBC, infiltrating tumor border configuration; pTBC, pushing tumor border configuration; mLN, MRI-detected metastatic lymph node; mTDs, MRI-detected tumor deposits; pTDs, pathology-proven tumor deposits; *: Fisher’s exact test.

### 3.2 Pathological results

Among the 114 patients with rectal cancer, 38 (33.3%) showed pLN, 13 (11.4%) showed postoperative distant metastases and 17 (14.9%) showed pathologically confirmed TDs. The 13 patients with postoperative distant metastases included 7 with liver metastases, 3 with lung metastases, 2 with simultaneous liver and lung metastases and 1 with iliac bone metastasis. Other pathological results are listed in **Tables 1** and **2**.

### 3.3 Univariate analysis

mTDs, mLN, iTBC, mEMVI, peritoneal reflection invasion, nodular protrusion at tumor edge, cord sign at the tumor edge and maximal extramural depth were significantly correlated with PDM and pLN (*P*<0.05) as shown in **Table 2**. Thirty (30/114, 26.3%) cases of mTDs were found, and among them 10 (10/30, 33.3%) developed PDM and 22 (22/30, 73.3%) developed lymph node metastasis. The maximal extramural depth was greater in both PDM-positive and pLN-positive groups than that in the correspondent negative group. mTDs and pTDs had moderate agreement (Kappa = 0.448). Thirty-six (36/114, 31.6%) cases of mLN were found, including 25 (25/36, 69.4%) from the pLN positive group, and mLN had a moderate agreement with pLN (Kappa = 0.520). A high degree of agreement occurred between the two radiologists in the assessment of mTDs (Kappa = 0.753).

### 3.4 Multiple logistic regression analysis and nomograms for PDM and pLN

**Figure 5:**
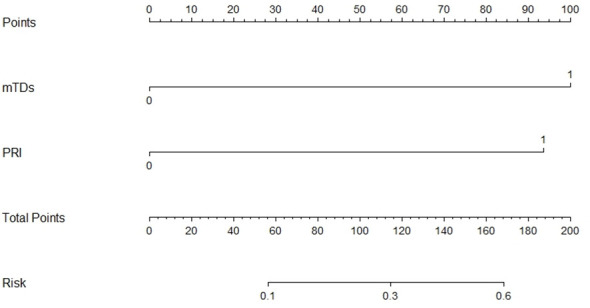
Nomogram for predicting PDM. The risk of developing postoperative distant metastases (PDM) of a patient with rectal cancer subjected only to MRI-detected tumor deposits (mTDs) and total points of 100 was approximately 26%. The total point was 193 (100 + 93), and the risk of PDM for this patient was 60% if this patient had both mTDs and peritoneal reflection invasion (PRI).

**Figure 6:**
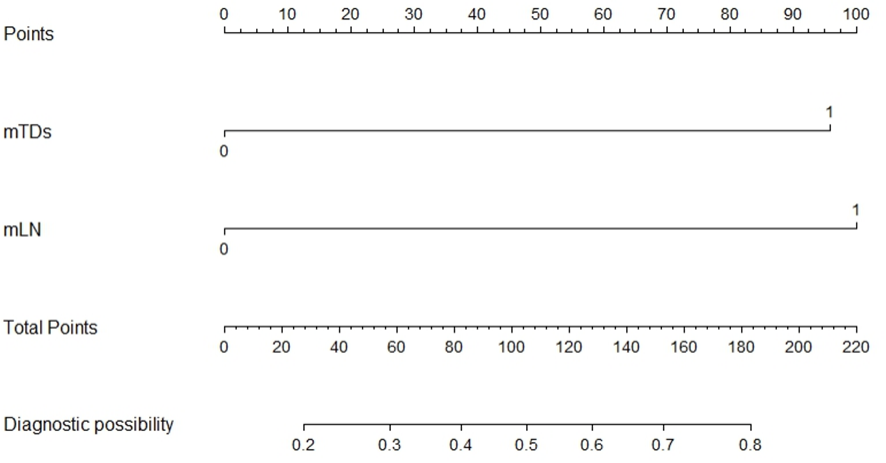
Nomogram for predicting pLN. The risk of developing pelvic lymph node metastasis of a patient with rectal cancer subjected only to MRI-detected lymph node involvement (mLN) and total points of 100 was approximately 48%. The total points were 196 (100 + 96), and the risk of pelvic lymph node metastasis for this patient was 80% if this patient had both MRI-detected tumor deposits (mTDs) and MRI-detected lymph node involvement (mLN).

**Figure 7:**
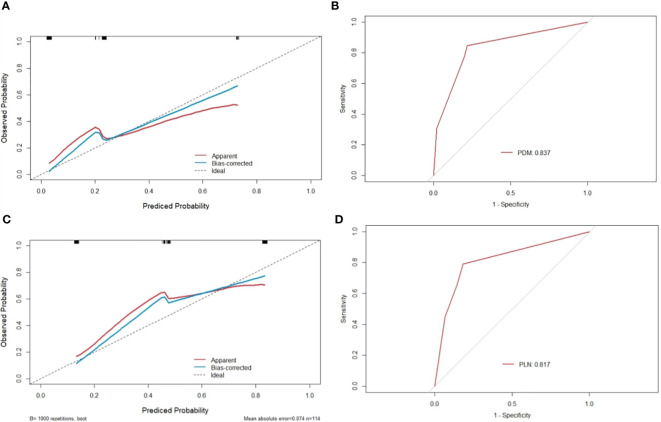
Calibration curves and ROC of nomograms for predicting PDM and pLN. **(A, C)** the x-axis represents the nomogram-predicted probability and the y-axis represents the actual probability of PDM and pLN. The perfect prediction corresponds to the 45° dotted line. The red solid line represents the entire cohort (n=114), and the blue solid line is the bias-corrected value by bootstrapping (B=1000 repetitions), indicating the observed nomogram performance. The calibration curve showed an evident relationship between the actual tag and the predicted tag. **(B)** the area under the ROC curve of the nomogram for predicting PDM was 0.837, suggesting that the confidence level of the probability of distant metastasis predicted by this nomogram was 83.7%. **(D)** the area under the ROC curve of the nomogram for predicting pLN was 0.817, suggesting that the confidence level of the probability of pelvic lymph node metastasis by this nomogram was 81.7%.

mTDs and peritoneal reflection invasion were independent predictors for PDM, as evaluated by multivariate logistic regression analysis, with odds ratios of 10.15 and 8.77, respectively. mTDs and mLN were independent predictors for pLN, with an odds ratio of 5.50 and 5.91, respectively (**Table 3**). The results of two models were not statistically significant, as revealed by the Hosmer-Lemeshow test, suggesting that the fit was good. Two nomograms were constructed to predict PDM and pLN according to the results of multivariate logistic regression analysis (**Figures 5**, **6**). The C-indices of the two nomograms were 0.837 and 0.817, respectively, after internal verification by bootstrap self-sampling. The calibration curves (**Figures 7A** **, C**) and ROC curves (**Figures 7B, D**) of the two nomograms showed that the preoperative predicted probability for PDM and pLN were well correlated with their actual incidence.

**Table 3:** Results of adjusted multivariate logistic regression analysis for MRI parameters of PDM and pLN.

Variable	PDM			aOR	pLN	P value
	aOR	95% CI	P value		95% CI	
mTDs	10.15	2.40-42.88	0.002	5.50	1.85-16.38	0.002
Peritoneal reflection invasion	8.77	1.61-47.72	0.012			0.566
mLN			0.143	5.91	2.12-16.50	0.001

aOR, adjusted odds ratio; 95% CI: 95% confidence intervals.

### 3.5 Predictive performance of MRI indicators and nomograms for PDM and pLN

**Figure 8:**
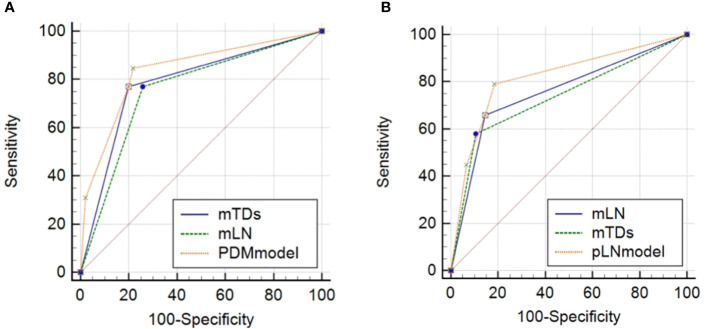
Comparisons of ROC curves of MRI indicators with nomograms for predicting PDM **(A)** and pLN **(B)**. mTDs, MRI-detected tumor deposits; mLN, MRI-detected metastatic lymph node; PDM, postoperative distant metastasis; pLN, pathology-proven lymph node involvement.

The AUC of mTDs for predicting PDM was 0.786 (95% CI: 0.645-0.926), with a sensitivity of 76.9%, a specificity of 80.2%, a positive predictive value of 33.3%, a negative predictive value of 96.4% and an accuracy of 79.8% (**Table 4**). The AUC of mLN for predicting pLN was 0.757 (95% CI: 0.655-0.858), with a sensitivity of 65.8%, a specificity of 85.5%, a positive predictive value of 69.4%, a negative predictive value of 83.3%, and an accuracy of 78.9% (**Table 4**). The sensitivities of the nomograms for predicting PDM and pLN were 84.6% and 78.9%, respectively, which were higher than all MRI indicators (**Table 4**). The DeLong test showed that the predictive efficacy of the nomogram for predicting pLN was better than that of mLN and mTDs (*P* = 0.0129 and 0.0221) (**Figure 8**).

**Table 4:** Predictive value of MRI indicators and nomogram in predicting PDM and pLN.

Model	Predictor	Sensitivity (%)	Specificity (%)	PPV (%)	NPV (%)	Accuracy (%)	AUC (95% CI)
PDM							
	mTDs	76.9	80.2	33.3	96.4	79.8	0.786 (0.645-0.926)
	mLN	76.9	74.3	27.8	96.2	74.6	0.756 (0.614-0.898)
	mEMVI	69.2	74.3	25.7	94.9	73.7	0.717 (0.564-0.871)
	Maximal extramural depth (>5mm)	84.6	70.3	26.8	97.3	71.9	0.775(0.647-0.902)
	Cord sign	76.9	68.3	23.8	95.8	69.3	0.726(0.583-0.870)
	Nodular protrusion	61.5	70.3	21.1	93.4	69.3	0.659(0.497-0.822)
	iTBC	76.9	63.4	21.3	95.5	64.9	0.701(0.556-0.847)
	Nomogram	84.6	78.2	33.3	97.5	78.9	0.837(0.720-0.954)
pLN							
	mTDs	57.9	89.5	73.3	81.0	78.9	0.737(0.631-0.842)
	mLN	65.8	85.5	69.4	83.3	78.9	0.757(0.655-0.858)
	mEMVI	55.3	81.6	60.0	78.5	72.8	0.684(0.575-0.793)
	Maximal extramural depth (>5mm)	60.5	76.3	56.1	79.5	71.1	0.684(0.577-0.791)
	Cord sign	57.9	73.7	52.4	77.8	68.4	0.658(0.549-0.767)
	Nodular protrusion	57.9	78.9	57.9	78.9	71.9	0.684(0.576-0.792)
	iTBC	65.8	71.1	53.2	80.6	69.3	0.684(0.578-0.790)
	Nomogram	78.9	81.6	68.2	88.6	80.7	0.817(0.736-0.898)

PDM, postoperative distant metastases; pLN, pathology-proven lymph node involvement; PPV, positive predictive value; NPV, negative predictive value; AUC, area under the ROC curve; mTDs, MRI-detected tumor deposition; PRI, peritoneal reflex invasion; mLN, MRI-detected lymph node involvement; mEMVI, MRI-detected extramural vascular invasion.

## 4 Discussion

Distant metastasis is the main cause of death in patients with colorectal cancer. However, metachronous distant metastasis is difficult to detect because it has a long latency period and it lacks clinical symptoms. Therefore, the assessment of effective means to predict metastasis may improve the prognosis of colorectal cancer. This work revealed that not only tumor deposition was detected by multiplanar HRT2WI, but also mTDs had a high predictive value for postoperative distant metastases and pelvic lymph node metastasis. Therefore, the screening of high-risk patients who are prone to postoperative metastasis might be beneficial for making personalized treatment plans according to our results.

Since the 20th century, isolated tumor nodules present in the pericolorectal adipose tissue or adjacent to the mesentery without clear vascular structures or lymph node components have been considered as TDs in colorectal cancer. They are small tumors in the adipose tissue outside the colon or rectum, unlike the metastatic lymph nodes (15, 16). For this reason, a slightly lower signal intensity was used on T2WI of the nodule that resembled the tumor as a criterion for the evaluation of mTDs. The origin of TDs was associated with venous invasion, lymphatic invasion, and neural infiltration (17, 18). Therefore, one of the most important aspect of our evaluation was to determine the location of the vessels in relation to the nodes by multiplanar HRT2WI. Our results revealed that mTDs were significantly associated with both postoperative distant metastases and lymph node metastasis, being a risk factor for both these poor prognostic factors. Patients with rectal cancer who developed mTDs had a 10.15 and 5.5-fold increased risk of distant metastases and lymph node metastasis, respectively, compared with their negative counterparts. Lord AC (8) recently showed a positive rate of 36% for mTDs, while our study found a positive rate of only 25.5% for mTDs. This difference could be due to differences in the characteristics of the samples and differences in the criteria for assessing mTDs. The mTDs in our study were mainly nodal foci with significantly irregular morphology in the pelvis, and also included a small number of round-like and oval-like cancer nodes, as well as some cancer nodes connected to the narrow base of the rectal cancer masses in the category of mTDs. Indeed, our results demonstrated that the narrow base connecting the nodes and the primary tumor was mostly made up of invading extramural vessels, and the border between the nodes and the primary tumor was recognizable. In addition, the presence of multiple fused nodules were considered as evaluation criteria as well, as well as more criteria for evaluation, which might have contributed to our slightly lower detection rate. The incidence of pTDs in the study of Lord AC was only 6%, and no concordance analysis was performed between mTDs and pTDs, while our study performed a concordance analysis between the two, revealing a moderate concordance that indicated that our MRI detection data for TDs were still relatively reliable. Other studies on pathological histology of TDs also showed relatively large differences in their incidence, ranging from 10.2% to 44.2%, with a median incidence of 21.3% (19), while our study discovered a low detection rate of pTDs of only 14.9%, which might be related to the detection method of the pathological histology, differences in the number of the sections, thickness of the sections, and pathologists’ understanding of TDs. Therefore, in addition to increasing the number of sections, special pathological techniques and methods might be required to improve the detection rate of TDs by pathological histology, while conventional pathological sections and observation methods might cause partial omissions. The low agreement between mTDs and pTDs in our study might be due to the fact that was not enough to rely only on the abnormal morphological and signal of nodules and the relationship with blood vessels, because the ability of MRI to discriminate tiny tumor foci, especially occult cancer nodules is very limited. Thus, the assessment of TDs by MRI still needs the introduction of new methods and techniques to continuously improve it, perhaps with the analysis by artificial intelligence in the future. However, the mTDs in our study still possessed a certain prognostic value, probably because the appearance of mTDs suggested that the tumor was poorly differentiated, highly invasive, and the surrounding tissues were easily invaded, while the TDs themselves often invade the adjacent blood vessels, lymphatic vessels and involve the rectal mesenteric fascia, causing distant metastases, which also increases the difficulty of complete removal of the tumor by surgery. The finding of mTDs as a risk factor for pLN in our study might also suggest that some of the mTDs originated from lymphatic vessel invasion.

Lymph node status plays a critical role in the choice of the treatment strategy to cure rectal cancer, and the presence or absence of pelvic lymph node metastasis influences the selection of the treatment. However, the assessment of metastatic lymph nodes by imaging currently remains a great challenge. Previous findings showed that the performance of MRI in detecting metastatic lymph nodes is usually good (14, 20). Currently, various diagnostic criteria are available to detect metastatic lymph nodes, including size, shape, border and signal alteration, while the only detection of the lymph node size does not improve the accuracy of lymph node staging in colorectal cancer (14, 20, 21), especially for very small nodes, which are very difficult to diagnose even with HRMRI due to the limitations of the spatial resolution and layer MRI thickness. In addition, the sizes of benign and malignant lymph nodes overlap. Therefore, mLN in our study was comprehensively assessed by combining the morphological size of the nodes, signal alteration, dynamic enhancement, and DWI, but the results were still not good, with only a moderate agreement between mLN and pLN, and the sensitivity of mLN was low. However, the nomogram for predicting lymph node metastasis constructed according to multi-factor logistic regression results showed good predictive performance with a good sensitivity and a large AUC, whose predictive performance was significantly better than that of mLN. Thus, the predictive model had a better efficacy in predicting metastatic lymph node, being meaningful for high-risk patients prone to lymph node metastasis, with certain clinical value.

Based on our daily clinical experience, we have observed that the presence of cord sign and nodular protrusions at the tumor edge is often associated with EMVI, distant metastasis, and regional lymph node metastasis. Therefore, these nodular protrusions and cord signs observed on MRI at the tumor edge may correspond to the clustered and cord-like tumor tissue at the tumor edge described in the pathological characterization of iTBC in the Jass study (11, 12). Consequently, we defined the presence of multiple irregular nodular protrusions and irregular cord-like features at the tumor edge observed on MRI as iTBC in our study.

Our study revealed a significant association between iTBC and lymph node metastasis as well as distant metastasis, which is consistent with the findings of Qwaider and Aboelnasr (22, 23) regarding the pathological features of iTBC. This suggests that the TBC detected by MRI has a certain degree of concordance with the histopathological iTBC. Furthermore, our recent study also demonstrated a significant correlation between nodular protrusions at the tumor edge and extramural venous invasion (EMVI) (24). Halvorsen revealed that patients with iTBC do not show evident peritumoral inflammation (25). In contrast, a dense inflammatory infiltrate is present in the peritumoral region of pTBC. The density of the inflammatory response around the tumor reflects the efficiency of the anti-tumor host response, which may represent one of the reasons why TBC has an impact on the prognosis of patients (26). These findings collectively suggest that iTBC is associated with various adverse prognostic indicators.

There have been limited previous reports on the correlation between peritoneal reflection invasion detected by MRI and metastasis. In our study, we observed a significant association between peritoneal reflection invasion and postoperative metastasis as well as pelvic lymph node metastasis in patients with rectal cancer. The presence of peritoneal reflection invasion in rectal cancer patients increased the risk of PDM by 8.77 times compared to patients without peritoneal reflection invasion. The invasion of the peritoneal reflection by the primary tumor suggests tumor penetration through the peritoneum and infiltration of tumor cells. These tumor cells can both proliferate at the invasion site and potentially detach and enter the pelvic cavity, leading to the development of multiple disseminated foci in the pelvis and other areas of the abdominal cavity. Consequently, surgical resection becomes more challenging, with a higher likelihood of distant metastasis. Furthermore, the presence of peritoneal reflection invasion indicates infiltration of tumor cells into the blood vessels, lymphatic vessels, and lymph nodes located between the peritoneal reflection and the anterior wall of the rectum. Therefore, rectal tumors with peritoneal reflection invasion may exhibit higher rates of tumor recurrence, EMVI, and lymph node metastasis, ultimately leading to poorer patient survival rates (27).

Despite the encouraging results, this work has also some limitations. Firstly, it was not possible to accurately compare MRI with histopathology because this work was a retrospective study. Secondly, our study was a single-center study with a small sample size unevenly distributed, especially for PDM, potentially leading to biased results, including large OR values. Therefore, our future plan is to add enough PDM for the analysis. Thirdly, the agreement between mTDs and pTDs in our study was not high, thus, the assessment criteria should be refined by expanding the sample, and designing a joint multidisciplinary prospective study. Artificial intelligence-assisted analysis should be also taken into consideration, since it could improve the detection rate and the diagnostic value of mTDs. Fourthly, the cords at the tumor margin in this study were defined only by morphology, while they might include desmoplastic reaction, invaded vessels, and cancerous lymphangitis, but a distinction was not made. However, all the above three characteristics had been confirmed as corresponding to a poor prognosis.

## 5 Conclusion

In summary, TDs can be detected by multiplane HRT2WI, and mTDs is an independent predictor for PDM and pelvic lymph node metastasis. The nomograms based on mTDs show a good predictive value for PDM and pelvic lymph node metastasis. These findings facilitate the preoperative selection of patients with high risk of distant metastases and lymph node metastases based on MRI. An appropriate personalized treatment strategy and follow-up can improve the prognosis of these patients.

## Data availability statement

The datasets presented in this study can be found in online repositories. The names of the repository/repositories and accession number(s) can be found in the article/supplementary material.

## Ethics statement

The studies involving humans were approved by Ethics Committee of Beijing Friendship Hospital. The studies were conducted in accordance with the local legislation and institutional requirements. Written informed consent for participation was not required from the participants or the participants’ legal guardians/next of kin in accordance with the national legislation and institutional requirements.

## Author contributions

BHL conceived and designed the analysis. Data collection was performed by XK, BHL, XJC and YLC. Analysis and interpretation of the data were performed by BHL and XK. EHJ critically revised the manuscript focusing on the important intellectual content. All authors contributed to the article and approved the submitted version.
